# Spectroscopic properties in Er^3+^-doped germanotellurite glasses and glass ceramics for mid-infrared laser materials

**DOI:** 10.1038/srep43186

**Published:** 2017-03-07

**Authors:** Shiliang Kang, Xiudi Xiao, Qiwen Pan, Dongdan Chen, Jianrong Qiu, Guoping Dong

**Affiliations:** 1 State Key Laboratory of Luminescent Materials and Devices and Guangdong Provincial Key Laboratory of Fiber Laser Materials and Applied Techniques, School of Materials Science and Engineering, South China University of Technology, Guangzhou 510640, China; 2 Key Laboratory of Renewable Energy, Guangdong Key Laboratory of New and Renewable Energy Research and Development, Guangzhou Institute of Energy Conversion, Chinese Academy of Sciences, Guangzhou 510640, China; 3 College of Optical Science and Engineering, State Key Laboratory of Modern Optical Instrumentation, Zhejiang University, Hangzhou 310027, China

## Abstract

Transparent Er^3+^-doped germanotellurite glass ceramics (GCs) with variable Te/Ge ratio were prepared by controllable heat-treated process. X-ray diffraction (XRD) and transmission electron microscope (TEM) confirmed the formation of nanocrystals in glass matrix. Raman spectra were used to investigate the evolution of glass structure and photon energy. Fourier transform infrared (FTIR) spectra were introduced to characterize the change of hydroxyl group (OH^−^) content. Enhanced 2.7 μm emission was achieved from Er^3+^-doped GCs upon excitation with a 980 nm laser diode (LD), and the influence of GeO_2_ concentration and heat-treated temperature on the spectroscopic properties were also discussed in detail. It is found that the present Er^3+^-doped GC possesses large stimulated emission cross section at around 2.7 μm (0.85 × 10^−20^ cm^2^). The advantageous spectroscopic characteristics suggest that the obtained GC may be a promising material for mid-infrared fiber lasers.

Recently, mid-infrared (MIR) lasers operating at around 3 μm have triggered increasing interest in coherent MIR light sources. This is mainly due to the strong absorption band by water at this wavelength and ~3 μm MIR lasers cover a great number of important molecular characteristic spectral lines, which make MIR fiber lasers appropriate in the fields of medical surgery, remote sensing, environmental monitoring and so forth^1^,^2^,^3^,^4^,^5^. Er^3+^ is an ideal emission candidate for 3 μm emission due to the transition of ^4^I_11/2_ → ^4^I_13/2_ and the absorption bands of Er^3+^ match well with the commercially available and low-cost 808 nm or 980 nm laser diode (LD)^6^. Among Er^3+^-doped materials, TeO_2_-based GCs have attracted great attention due to the following advantages. On one hand, GCs combine the advantages of excellent fiber-drawing ability of glass^7^,^8^ and strong crystal field of nanocrystals^9^,^10^. On the other hand, tellurite glasses possess wide transmission region (from 0.35 to 5 μm), the lowest phonon energy (~750 cm^−1^) among the common oxide glasses, large refractive index (~2.0), high solubility for rare earth ions and good chemical durability etc^11^,^12^,^13^,^14^. The low phonon energy is very beneficial to the 2.7 μm emission of Er^3+^ ions due to the narrow gap between ^4^I_11/2_ and ^4^I_13/2_ levels. The large refractive index enhances emission cross section and improves the refractive index match between nanocrystals and glass matrix.

The successful fabrication of a new cubic crystalline phase in K_2_O-Nb_2_O_5_-TeO_2_ glasses in 1995 and the investigation on structure in TeO_2_-PbF_2_-CdF_2_ GCs in 2002 have triggered great interests in tellurite GCs^15^,^16^. Although a few studies have been conducted on tellurite GCs, to our best knowledge, no work is reported about the 2.7 μm emission of Er^3+^-doped germanotellurite glasses and GCs system. Not to mention the effect of GeO_2_ concentration on the spectroscopic properties of Er^3+^-doped germanotellurite glasses. In this work, we obtained enhanced 2.7 μm emission from Er^3+^-doped germanotellurite glasses and GCs, and the influence of GeO_2_ concentration and heat-treated temperature on the spectroscopic properties of Er^3+^-doped germanotellurite glasses and GCs are discussed in detail.

## Results and Discussion

### The effect of Te/Ge ratio on the spectroscopic properties

**Figure 1:**
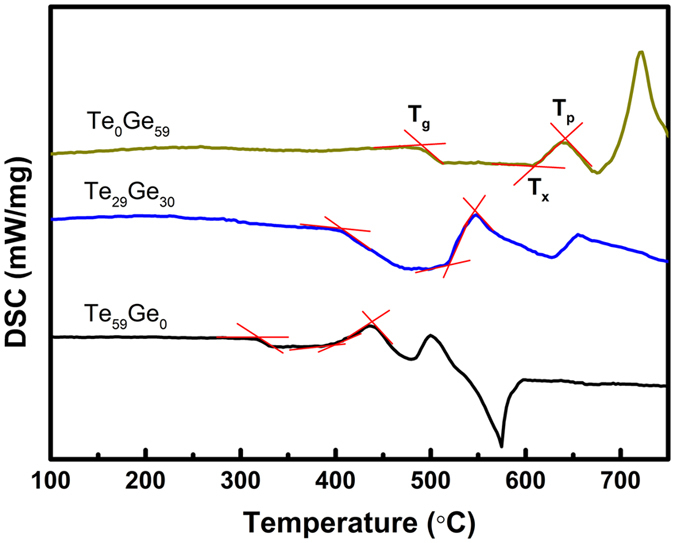
DSC curves of Te_(59−x)_Ge_x_ (x = 0, 30, 59) glasses.

In order to analyze the effect of GeO_2_ concentration on the thermal property, DSC curves of representative Te_59_Ge_0_, Te_29_Ge_30_, and Te_0_Ge_59_ samples are measured and shown in Fig. 1. We can observe that the glass transition temperature (T_g_), onset crystallization temperature (T_x_), and crystallization peak temperature (T_p_) increase significantly with the increase of GeO_2_ concentration. It can be ascribed to the fact that GeO_2_ acts as a network former and can collect glass network and strengthen the structure of the network^17^. In order to evaluate the thermal stability of prepared samples, the parameter ∆T (T_x_ − T_g_) is obtained, which is usually used to estimate the glass stability. It can be seen from Table 1 that the values of ∆T increases with the increase of GeO_2_ concentration. A large ∆T means the strong inhibition of nucleation and crystallization.

**Figure 2:**
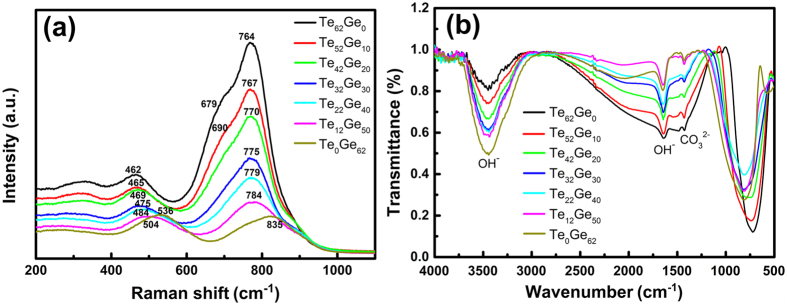
(**a**) Raman spectra, and (**b**) FTIR spectra of undoped Te_(62−x)_Ge_x_ (x = 0~62) glasses.

In order to investigate the evolution of glass structure with the component variation, Raman spectra are carried out on precursor glasses. Figure 2(a) shows the Raman spectra of undoped precursor glasses excited by a 532 nm laser. The peak located at 462 cm^−1^ can be attributed to the stretching mode of Te-O-Te symmetric bridges, the shoulder about 679 cm^−1^ is identified as the stretching vibration of Te-O bonds in the TeO_4_ trigonal bipyramid, and another peak located at 764 cm^−1^ is assigned to the stretching vibration of Te-O bond in TeO_3+1_ or TeO_3_ units^17^,^18^. It is known that the intensities and the positions of the Raman peaks depend on the concentration and kind of structural groups. As the GeO_2_ concentration increases, the peaks at 462 cm^−1^ and 764 cm^−1^ gradually shift toward higher frequencies, and at the same time the intensity of the peak at 764 cm^−1^ decreases. This behavior is due to the formation of GeO_4_ units and a part of TeO_3+1_ or TeO_3_ units are replaced by GeO_4_ units. One can observe that when TeO_2_ is absent, the peaks shifted to 536 cm^−1^ and 835 cm^−1^, which corresponds to the symmetric stretching and asymmetric stretching of Ge-O-Ge bridges in GeO_4_ tetrahedra, respectively^19^,^20^. Additionally, as the location of the Raman peak is relevant with the phonon energy of glass, the addition of GeO_2_ leads to an increase of the phonon energy of glass.

To clarify the influence of GeO_2_ concentration on the content of OH^−^ in glass, the FTIR spectra of precursor glasses are recorded in Fig. 2(b). The bands at around 1636 cm^−1^ and 3439 cm^−1^ are belong to the bending vibration and stretching vibration of the OH^−^ group, respectively. The band at approximately 1423 cm^−1^ can be assigned to the stretching vibration of the 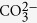 group^21^,^22^. It can be easily observed that the content of OH^−^ increases gradually with the increase of GeO_2_ concentration. On the basis of electronegativity theory, covalency decreases with the difference of electronegativity between cation and anion ions. The values of electronegativity for Te, Ge, and O elements are about 2.1, 1.8, and 3.5, respectively^23^. Since the larger electronegativity difference between Ge and O indicates weaker covalency of Ge-O bond, the ionicity of Ge-O bond is stronger than that of Te-O bond. Therefore, Ge^4+^ will absorb more OH^−^ group, which results in the higher content of OH^−^ with the increase of GeO_2_ concentration. The bands in the range from 500 to 1200 cm^−1^ are assigned to the vibrations of glass network former, and the locations of the peaks shift to longer wavenumber with the addition of GeO_2_ content, which matches well with the result of Raman spectra as mentioned before.

**Figure 3:**
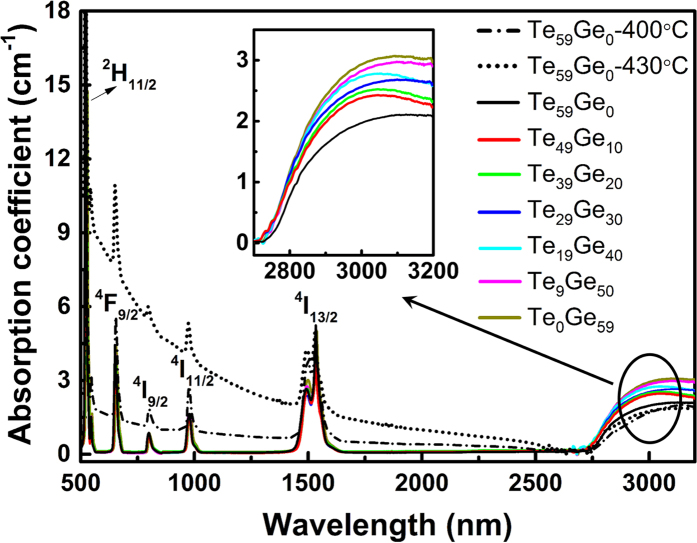
Absorption spectra of 3% Er^3+^-doped Te_(59−x)_Ge_x_ (x = 0~59) glasses and Te_59_Ge_0_ GCs heat-treated at different temperature for 8 h. The inset shows the enlarged image of absorption spectra in the range from 2700 nm to 3200 nm.

Figure 3 shows the absorption spectra of precursor glasses and GCs in the range of 500–3200 nm. Five absorption bands at 524 nm, 653 nm, 800 nm, 978 nm and 1535 nm are found and attributed to the transitions of Er^3+^ ions from the ground state ^4^I_15/2_ to the excited states ^2^H_11/2_, ^4^F_9/2_, ^4^I_9/2_, ^4^I_11/2_ and ^4^I_13/2_, respectively. The shape and peak positions of each transition remain unchanged with the increase of GeO_2_ concentration. The inset in Fig. 3 shows the enlarged image of absorption spectra of germanotellurite glasses in the range from 2700 nm to 3200 nm. It can be clearly observed that the absorption at around 3 μm corresponding to the stretching vibrations of -OH group increases monotonically with increasing GeO_2_ content, which can also be explained by electronegativity theory mentioned above. Obvious absorption peaks around 980 nm indicates that the prepared samples can be efficiently pumped by a commercial 980 nm LD.

According to absorption spectra, Judd-Ofelt parameters Ω_λ_ (λ = 2, 4, 6) can be determined by J-O theory, which have been successfully used to calculate 4 f electrons transitions of RE ions in different host materials^24^,^25^. Based on J-O theory, Ω_2_ is closely related to the covalency between RE ions and ligand anions, which reflects the asymmetry of local environment around the site of RE ions. The larger the Ω_2_ is, the stronger the covalency between RE ions and ligand anions becomes. Table 2 shows the J-O parameters of Er^3+^ in different samples. Based on the electronegativity theory mentioned above, the covalency of the Er-O bond increases with the increase of GeO_2_ concentration. As a result, the values of the parameter Ω_2_ show an obvious increase with increasing GeO_2_ content.

**Figure 4:**
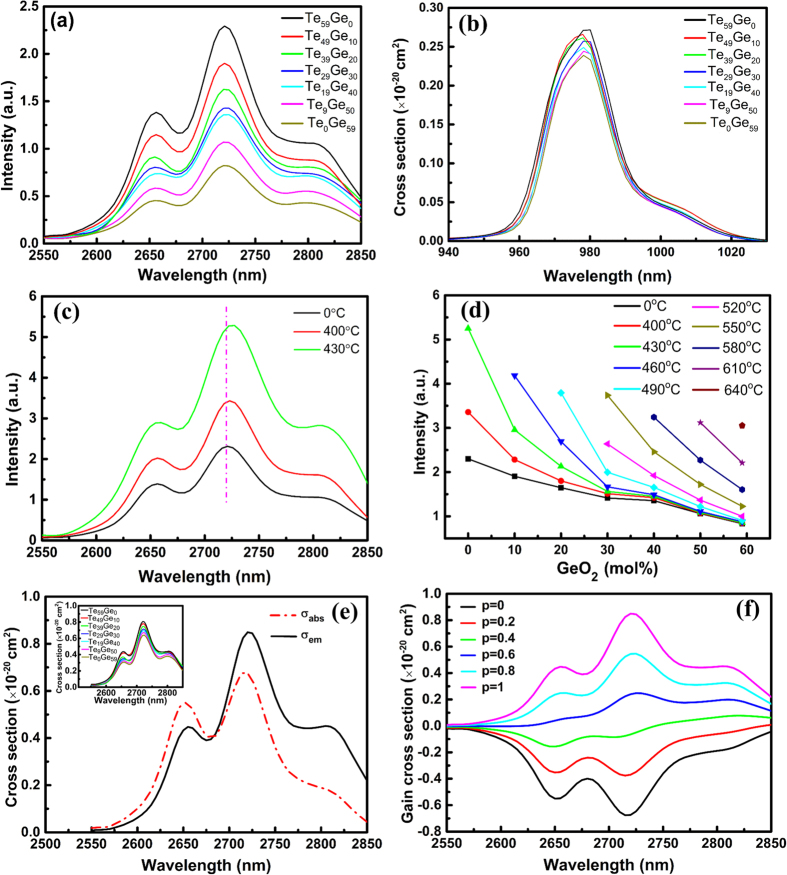
(**a**) 2.7 μm emission spectra, and (**b**) Absorption cross section at 980 nm of 3% Er^3+^-doped Te_(59−x)_Ge_x_ (x = 0~59) glasses (**c**) 2.7 μm emission spectra of 3% Er^3+^-doped Te_59_Ge_0_ glass and GCs heat-treated at 400 °C–430 °C for 8 h. (**d**) Composition and heat-treated temperature dependence integrated intensity of 2.7 um emission peaks; (**e**) Absorption and emission cross sections, and (**f**) gain cross sections at 2.7 μm in 3% Er^3+^-doped Te_59_Ge_0_ GC heat-treated at 430 °C for 8 h. The inset in figure (**e**) shows the emission cross sections of 3% Er^3+^-doped Te_(59−x)_Ge_x_ (x = 0~59) glasses.

Figure 4(a) shows the MIR emission spectra of Er^3+^-doped germanotellurite glasses under a 980 nm LD excitation. Obvious 2.7 μm emission peaks corresponding to Er^3+^: ^4^I_11/2_ → ^4^I_13/2_ transition can be observed. It is found that the 2.7 μm emission intensity decreases with the increase of GeO_2_ concentration. It is known that the intensity of MIR emission is very sensitive to the multiphonon relaxation rate of RE ions which strongly depends on the phonon energy of their matrix. Usually, the lower the phonon energy of host matrix is, the smaller the multiphonon relaxation probability is. In addition, the OH^−^ impurity in glasses can absorb and quench 2.7 μm emission signals, which is disadvantage for MIR emission. From the previous results, both the content of OH^−^ and the phonon energy of glass increase with the increase of GeO_2_ concentration. As a result, the 2.7 μm emission intensity decreases with the increasing GeO_2_ content. Based on the absorption spectra, the absorption cross section (*σ*_*abs*_) at 980 nm can be defined as^26^:


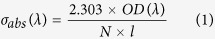


where OD(λ), N, and l represent the optical density, Er^3+^ doping concentration and sample thickness, respectively. The absorption cross section at 980 nm have been calculated and displayed in Fig. 4(b). It can be obtained that the peak absorption cross sections decrease with increasing GeO_2_ concentration. Higher absorption cross section is beneficial to the 2.7 μm emission. In order to estimate the 2.7 μm emission properties for potential laser applications, the emission cross sections at 2.7 μm in Er^3+^-doped germanotellurite glasses are calculated by the Fuchtbauer-Ladenburg equation^27^ and shown in the inset of Fig. 4(e). The detailed calculation processes can refer to ref. 14. We observe that the peak emission cross section decreases with the increasing GeO_2_ content.

**Figure 5:**
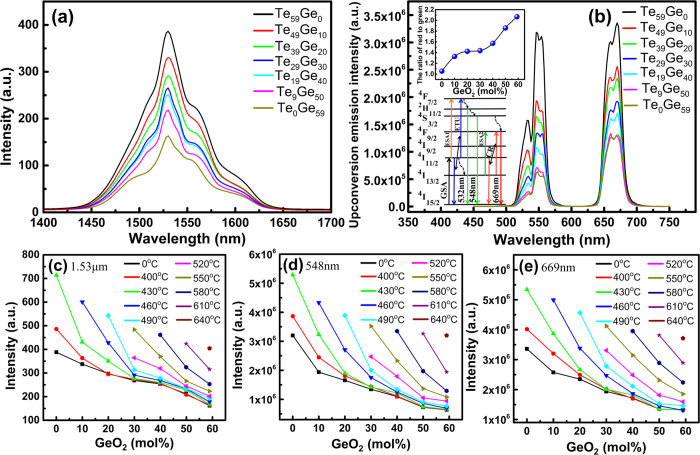
(**a**) 1.53 μm NIR and (**b**) UC emission spectra of 3% Er^3+^-doped Te_(59−x)_Ge_x_ (x = 0~59) glasses pumped by a 980 nm LD; (**c**–**e**) Composition and heat-treated temperature v.s. integrated intensity of 1.53 um, 548 nm and 669 nm emission peaks, respectively. The inset in figure (**b**) shows the GeO_2_ concentration dependence the ratio of red to green emission peaks and the energy level diagram of Er^3+^ under 980 nm LD excitation.

Figure 5(a,b) display the NIR and UC emission spectra of Er^3+^-doped germanotellurite glasses, respectively. It can be found that enhanced 1.53 μm emission corresponding to Er^3+^: ^4^I_13/2_ → ^4^I_15/2_ transition and 532 nm, 548 nm, and 669 nm emission corresponding to Er^3+^: ^2^H_11/2_ → ^4^I_15/2_, ^4^S_3/2_ → ^4^I_15/2_ and ^4^F_9/2_ → ^4^I_15/2_ transitions are observed. The NIR and UC emission intensity decrease with the increase of GeO_2_ concentration. The inset in Fig. 5(b) shows the ratio of red to green emission peaks in Er^3+^-doped glasses with different GeO_2_ concentration and the ratio increases gradually with the increase of GeO_2_ concentration. This can be explained by the energy level diagram of Er^3+^ shown in Fig. 5(b). For the green emission, electrons at ^4^I_15/2_ level are excited to ^4^I_11/2_ level through ground state absorption (GSA) under 980 nm LD pumping, then the excited electrons at ^4^I_11/2_ level are excited further to ^4^F_7/2_ level through excited state absorption (ESA1) or energy transfer upconversion process (ETU: ^4^I_11/2_ + ^4^I_11/2_ → ^4^I_15/2_ + ^4^F_7/2_). The electrons of ^4^F_7/2_ level decay nonradiatively to the next lower ^2^H_11/2_ and ^4^S_3/2_ levels, and the ^2^H_11/2_ → ^4^I_15/2_, ^4^S_3/2_ → ^4^I_15/2_ transitions produce green emissions. The red emission is originated from the ^4^F_9/2_ → ^4^I_15/2_ transition. The excited electrons at ^4^I_13/2_ level are excited to ^4^F_9/2_ level through excited state absorption (ESA2) or the electrons at ^4^S_3/2_ level decay nonradiatively to ^4^F_9/2_ level, then the ^4^F_9/2_ → ^4^I_15/2_ transition gives rise to red emission. It can be obtained from the energy level diagram of Er^3+^ that the electrons of ^4^F_7/2_ and ^4^F_9/2_ levels are primarily dominated by the ^4^I_11/2_ and ^4^I_13/2_ levels, respectively. As we all know, the quite narrow energy gap between ^4^I_11/2_ and ^4^I_13/2_ levels makes the nonradiative relaxation in ^4^I_11/2_ level remarkable for hosts with large phonon energy. While due to the relatively larger energy gap between ^4^I_13/2_ and ^4^I_15/2_ levels than that between ^4^I_11/2_ and ^4^I_13/2_ levels, the influence of matrix phonon energy on the nonradiative relaxation in ^4^I_13/2_ level is relatively small. Therefore, the nonradiative relaxation rate from ^4^I_11/2_ level becomes larger than that from ^4^I_13/2_ level with the increase of GeO_2_ concentration. In addition, cross relaxation process (CR: ^4^I_11/2_ + ^4^I_13/2_ → ^4^F_9/2_ + ^4^I_15/2_) also makes a contribution to the red emission. As a result, the ratio of red to green emission peaks gradually grows with increasing GeO_2_ concentration.

**Figure 6:**
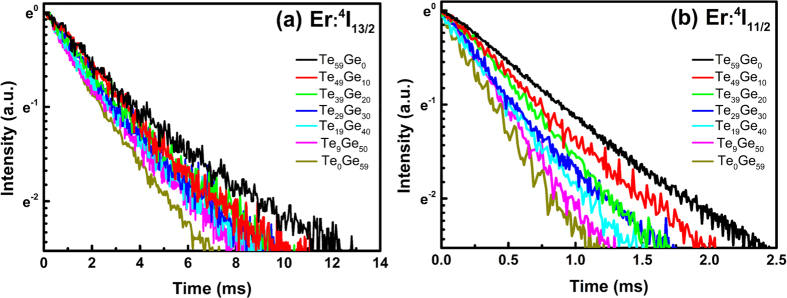
Fluorescence decay curves of (**a**) Er^3+^: ^4^I_13/2_ level pumped by pulsed 980 nm LD, and (**b**) Er^3+^: ^4^I_11/2_ level pumped by pulsed 808 nm LD in 3% Er^3+^-doped Te_(59-x)_Ge_x_ (x = 0~59) glasses.

Figure 6 reveals the fluorescence decay curves of Er^3+^: ^4^I_13/2_ level (Fig. 6(a)) and ^4^I_11/2_ level (Fig. 6(b)) excited by 808 and 980 nm LD as a function of GeO_2_ concentration. The fluorescence lifetime of a rare-earth ion in an excited state is mainly determined by the radiative decay rate, multiphonon decay rate, and energy transfer rate. Since the concentration of Er^3+^ ions in all of the samples is the same, the energy transfer rate of Er^3+^ ions should be close for all of the glass samples. Therefore, the measured lifetime of Er^3+^: ^4^I_13/2_ level and ^4^I_11/2_ level in glasses is mainly determined by the radiative decay rate and the multiphonon decay rate. In addition, the non-single exponential shape of the decay curves of Er^3+^: ^4^I_13/2_ level is probably associated to energy transfer. Table 3 shows the lifetimes of Er^3+^: ^4^I_13/2_ and Er^3+^: ^4^I_11/2_ levels for different samples. It can be seen that both the fluorescence lifetime of Er^3+^: ^4^I_13/2_ level and Er^3+^: ^4^I_11/2_ level decrease with the increase of GeO_2_ concentration. The content of OH^−^ and phonon energy of germanate glasses are higher than that of tellurite glasses, which can be confirmed by the FTIR and Raman spectra. Thus, the introduction of GeO_2_ to tellurite glass will lead to a decrease for the lifetime of Er^3+^: ^4^I_13/2_ level and ^4^I_11/2_ level by increasing the content of OH^−^ and phonon energy of glass, which will subsequently increase the multiphonon decay rate of Er^3+^ in glass samples. Therefore, it can be concluded that multiphonon decay rate has a dominant influence on the lifetime of Er^3+^: ^4^I_13/2_ level and ^4^I_11/2_ level for the glass samples.

### The effect of heat-treated process on the spectroscopic properties

**Figure 7:**
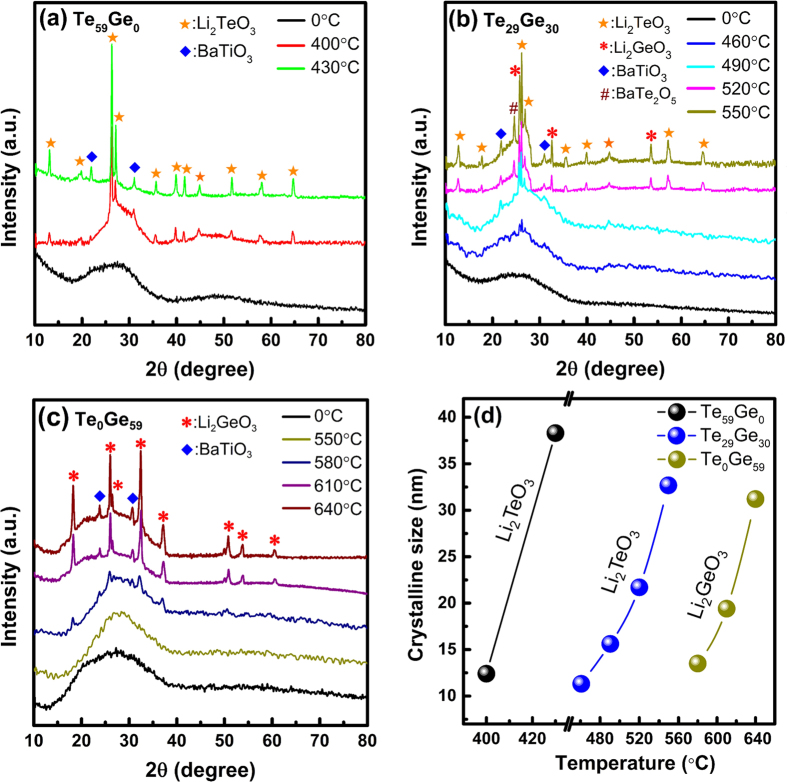
(**a**–**c**) XRD patterns of 3% Er^3+^-doped Te_59_Ge_0_, Te_29_Ge_30_, and Te_0_Ge_59_ glasses and GCs heat-treated at different temperature for 8 h. (**d**) the dependence of crystalline size on heat-treated temperature.

The XRD patterns of representative Te_59_Ge_0_, Te_29_Ge_30_, and Te_0_Ge_59_ samples heat-treated at different temperature are shown in Fig. 7(a,b and c), respectively. The precursor glasses are completely amorphous with no diffraction peaks. After a heat-treated process, several diffraction peaks, a characteristic of crystalline structure, are clearly observed, which are assigned to Li_2_TeO_3_ (JCPDF: 26–1192) and BaTiO_3_ (JCPDF: 34–0129) for (a), Li_2_TeO_3_ (JCPDF: 26–1192), Li_2_GeO_3_ (JCPDF: 34–0659), BaTiO_3_ (JCPDF: 34–0129) and BaTe_2_O_5_ (JCPDF: 36–0886) for (b), Li_2_GeO_3_ (JCPDF: 34–0659) and BaTiO_3_ (JCPDF: 34–0129) for (c). With the increase of heat-treated temperature, the diffraction peaks become more evident and sharper, which indicates crystalline size grows gradually. What is more, the crystallization is a bulk process (Supplementary Fig. S1).

From the peak width of XRD pattern, the crystalline size of samples can be calculated on the basis of the Scherrer’s equation^28^:


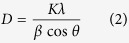


where *D* is the grain size of nanocrystals, *K* is a dimensionless shape factor, *λ* is the X-ray wavelength, *β* is the full-width at half maximum (FWHM) of the diffraction peak and *θ* is the Bragg angle. The diffraction peaks at 2θ = 26.17° in (a) and (b), and 2θ = 32.35° in (c) are used to obtain the crystalline size of Li_2_TeO_3_ and Li_2_GeO_3_ nanocrystal, respectively. The calculated crystalline size dependent on heat-treated temperature is plotted in Fig. 7(d). It can be seen that the crystalline size increases monotonically with increasing heat-treated temperature.

**Figure 8:**
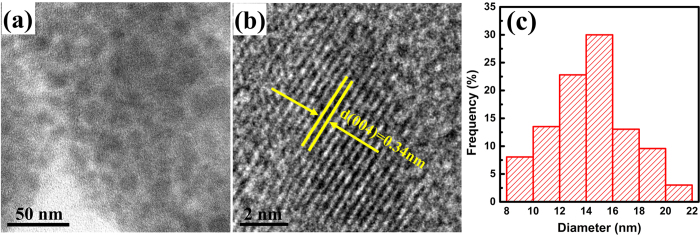
(**a**) TEM micrograph, (**b**) HRTEM image, and (**c**) size distribution histogram of 3% Er^3+^-doped Te_59_Ge_0_ GC heat-treated at 400 °C for 8 h.

Figure 8(a) shows the TEM image of GC-Te_59_Ge_0_ heat-treated at 400 °C for 8 h. The quasi-spherical particles dispersed in glass matrix homogeneously. The high-resolution transmission electron microscope (HRTEM) image in Fig. 8(b) displays the fine lattice structure with corresponding interplanar spacing about 0.34 nm, which can be ascribed to the (004) plane of Li_2_TeO_3_ nanocrystal. The particle size distribution histogram shown in Fig. 8(c) suggests that the particle size is distributed with diameters in the range of 8–20 nm with an average size of ~14 nm, which is in good agreement with our calculated result based on the XRD data.

The absorption spectra of Te_59_Ge_0_ GCs in the range of 500–3200 nm are displayed in Fig. 3. It can be seen that the baselines of the absorption curves become more precipitous toward to the shorter wavelength region with the increase of heat-treated temperature. It can be ascribed to the transmittance loss causing by the Rayleigh scatting effect, which is resulted from the growth of nanocrystals. According to absorption spectra, the J-O parameters of Er^3+^ in Te_59_Ge_0_ GCs are calculated and shown in Table 2. Based on J-O theory, the parameter Ω_2_ is sensitive to the environmental configuration symmetry of RE ions, and it decreases with the increase of heat-treated temperature, which indicates the variation of RE ions environment from glass to GCs. It can be seen from Table 2 that the value of Ω_2_ decreases with the increase of heat-treated temperature confirming the incorporation of Er^3+^ into the precipitated nanocrystals. In addition, the value of Ω_6_, which is proportional to the rigidity of the host, increases slightly in the transition from glass to GCs^29^.

Figure 4(c) shows the MIR emission spectra of Te_59_Ge_0_ glass and GCs under a 980 nm LD excitation. It is found that the 2.7 μm emission intensity enhances remarkably with the increase of heat-treated temperature. Moreover, a slight red shift of emission peak emerges in the GCs compared with the precursor glass, which is mainly due to the crystal field effect. Figure 4(d) shows the contrast of 2.7 μm emission intensity produced by Er^3+^-doped glasses with different heat-treated temperature and GeO_2_ concentrations. The 2.7 μm emission intensity enhances with the increase of heat-treated temperature under the condition of the same GeO_2_ concentration. According to the XRD pattern, the nanocrystals have better crystallinity at a higher temperature, inducing the enhancement the crystal field effect. Therefore, the 2.7 μm emission intensity enhances significantly with increasing crystallization temperature.

Figure 4(e) displays the absorption and emission cross sections at 2.7 μm in Er^3+^-doped tellurite GC heat-treated at 430 °C for 8 h. The peak absorption and emission cross sections in the present GC are 0.68 × 10^−20^ cm^2^ and 0.85 × 10^−20^ cm^2^, respectively. The obtained σ_em_ for present sample is higher than those of Er^3+^-doped bismuthate (0.77 × 10^−20^ cm^2^), germanate (0.70 × 10^−20^ cm^2^) glass and oxyfluoride GC (0.43 × 10^−20^ cm^2^), but smaller than the GC containing SrF_2_ crystals (1.20 × 10^−20^ cm^2^)^30^,^31^,^32^,^33^. Higher emission cross section is benefit for higher possibility to achieve laser output. In addition, Te_59_Ge_0_ glass still has a good transparency after heat treatment (Supplementary Fig. S2). The gain cross section, which is an important parameter to evaluate the gain performance of prepared sample, can be calculated by the following equation^34^:


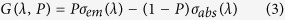


where P is the population inversion given by the ratio between the concentration of the Er^3+^: ^4^I_11/2_ level and the total Er^3+^ concentration. The gain cross sections for different P values are shown in Fig. 4(f). It is found that the gain becomes positive when P is more than 0.4 implying that a low pumping threshold can be achieved for the Er^3+^: ^4^I_11/2_ → ^4^I_13/2_ laser operation.

Figure 5(c–e) show the composition and heat-treated temperature dependent integrated intensity of 1.53 μm, 548 nm and 669 nm emission peaks in Er^3+^-doped germanotellurite GCs. It is clear shown that all the emission intensity increase with the increasing heat-treated temperature in the case of the same GeO_2_ concentration. These results exhibit similar trend with MIR emission, which can be explained by the similar crystal field effect mentioned above.

## Conclusions

In summary, Er^3+^-doped germanotellurite glasses and GCs are prepared and the structural and spectroscopic properties are investigated. From FTIR and Raman spectra, it is noted that the addition of GeO_2_ increases the content of OH^−^ and phonon energy of glasses. J-O intensity parameters are calculated and discussed. Moreover, the influence of GeO_2_ concentration and heat-treated temperature on the MIR, NIR and UC emissions are discussed in detail. Enhanced 2.7 μm emission is observed in Er^3+^-doped germanotellurite GCs and the tellurite GC heat-treated at 430 °C for 8 h possesses large absorption (0.68 × 10^−20^ cm^2^) and emission (0.85 × 10^−20^ cm^2^) cross sections for Er^3+^: ^4^I_11/2_ → ^4^I_13/2_ transition. These results imply that this GC might be a promising candidate for Er^3+^-doped MIR fiber lasers.

## Methods

### Preparation of Er^3+^-doped GCs

Germanotellurite glasses with molar compositions of (59-*x*) TeO_2_-*x* GeO_2_-8 TiO_2_-8 BaO-22 Li_2_O-3 Er_2_O_3_ (x = 0, 10, 20, 30, 40, 50, 59), which denoted as Te_59_Ge_0_, Te_49_Ge_10_, Te_39_Ge_20_, Te_29_Ge_30_, Te_19_Ge_40_, Te_9_Ge_50_, and Te_0_Ge_59_ correspondingly, were prepared by the conventional melt-quenching method. TeO_2_ (5N), GeO_2_ (5N), TiO_2_ (4N), BaCO_3_ (A.R.), Li_2_CO_3_ (A.R.), and Er_2_O_3_ (4N) were used as raw materials and each batch of 20 g was well-mixed and melted in a covered corundum crucible at 1100 °C for 40 min at ambient temperature. The melt was cast onto a preheated copper plate and then cooled down to room temperature. The glass samples were cut to the size of 10 mm × 10 mm and heat-treated at different temperature for 8 h to achieve GCs. Then the samples were polished to optical quality before measurements with a thickness of 1.5 mm.

### Characterization

Differential scanning calorimetry (DSC) was performed in a simultaneous thermal analyzer (STA449C NETZSCH) under N_2_ atmosphere with heating rate at 10 °C /min in order to investigate the thermal property and determine the crystallization characteristic temperatures. XRD measurements were carried out on a X’Pert PRO X-ray diffractometer by using Cu Kα_1_ as a radiation source to identify the crystalline phase and estimate the nanocrystal grain size. Transmission electron microscope (TEM, 2100 F, JEOL, Japan) was performed to analyze the micrograph and microstructure of GC sample. Raman spectra were recorded in the range of 200–1100 cm^−1^ using a Raman spectrometer (Renishaw in Via, London, UK) with an excitation of 532 nm laser. The FTIR spectra were obtained on a Vector 33 Fourier transform infrared spectrophotometer (Bruker, Switzerland) to estimate the content of OH^−^ in glasses. The absorption spectra were measured in the range of 500–3200 nm using a Perkin-Elmer Lambda 900/UV/VIS/NIR spectrophotometer. The mid-infrared (MIR) emission spectra in the range of 2550–2850 nm were recorded by a spectrometer (zolix, Omni 5015i, Beijing, China) with a lock-in amplifier upon excitation of a 980 nm LD. The near-infrared (NIR) and upconversion (UC) emission spectra pumped by 980 nm LD were measured on a computer controlled Triax 320 type spectrofluorimeter (Jobin-Yvon Corp.). The fluorescence decay curves of Er^3+^: ^4^I_11/2_ and ^4^I_13/2_ levels were captured by a Tektronix TDS 3012c Digital Phosphor Oscilloscope with pulsed 808 and 980 nm LDs as excitation sources, respectively. All the measurements were carried out at room temperature.

## Additional Information

**How to cite this article**: Kang, S. *et al*. Spectroscopic properties in Er^3+^-doped germanotellurite glasses and glass ceramics for mid-infrared laser materials. *Sci. Rep.* **7**, 43186; doi: 10.1038/srep43186 (2017).

**Publisher's note:** Springer Nature remains neutral with regard to jurisdictional claims in published maps and institutional affiliations.

## Supplementary Material

Supplementary Information

## Figures and Tables

**Table 1:** The temperatures of glass transition (T_g_), onset crystallization (T_x_), peak crystallization (T_p_), and thermal stability parameter ∆T in various glasses.

Samples	T_g_ (°C)	T_x_ (°C)	T_p_ (°C)	∆T (°C)
Te_59_Ge_0_	315	396	437	81
Te_29_Ge_30_	411	516	548	105
Te_0_Ge_59_	493	607	641	114

**Table 2:** J-O intensity parameters of Er^3+^ in different samples.

Samples	Ω_2_ (×10^−20^ cm^2^)	Ω_4_ (×10^−20^ cm^2^)	Ω_6_ (×10^−20^ cm^2^)	δ (×10^−6^)
Te_59_Ge_0_	5.03 ± 0.03	1.47 ± 0.04	1.19 ± 0.04	0.15
Te_49_Ge_10_	5.54 ± 0.04	1.42 ± 0.01	1.09 ± 0.06	0.17
Te_39_Ge_20_	5.71 ± 0.03	1.61 ± 0.07	1.12 ± 0.04	0.14
Te_29_Ge_30_	6.03 ± 0.04	1.83 ± 0.03	1.04 ± 0.10	0.23
Te_19_Ge_40_	6.33 ± 0.05	1.72 ± 0.07	0.95 ± 0.08	0.27
Te_9_Ge_50_	6.76 ± 0.08	1.79 ± 0.03	0.92 ± 0.04	0.19
Te_0_Ge_59_	7.04 ± 0.06	1.93 ± 0.05	0.80 ± 0.11	0.17
Te_59_Ge_0_-400 °C	4.90 ± 0.02	1.45 ± 0.06	1.21 ± 0.06	0.14
Te_59_Ge_0_-430 °C	4.67 ± 0.06	1.41 ± 0.04	1.22 ± 0.04	0.13

**Table 3:** The lifetimes of Er^3+^: ^4^I_13/2_ and Er^3+^: ^4^I_11/2_ levels for different samples.

Samples	τ (^4^I_13/2_) (ms)	τ (^4^I_11/2_) (ms)
Te_59_Ge_0_	3.42 ± 0.031	0.89 ± 0.007
Te_49_Ge_10_	3.07 ± 0.004	0.75 ± 0.009
Te_39_Ge_20_	2.86 ± 0.019	0.64 ± 0.008
Te_29_Ge_30_	2.70 ± 0.017	0.54 ± 0.011
Te_19_Ge_40_	2.61 ± 0.009	0.49 ± 0.011
Te_9_Ge_50_	2.46 ± 0.013	0.45 ± 0.009
Te_0_Ge_59_	2.32 ± 0.007	0.40 ± 0.010
