# Supplementary material for: Spectroscopic properties in Er3+-doped germanotellurite glasses and glass ceramics for mid-infrared laser materials
Source: Sci Rep. 2017 Mar 7;7:43186. doi: 10.1038/srep43186 (PMC5339781; doi:10.1038/srep43186)
Supplement: Supplementary Information [file srep43186-s1.pdf]

Supporting information

Spectroscopic properties in Er^{3+}-doped germanotellurite glasses and glass ceramics for mid-infrared laser materials

Shiliang Kang[1], Xiudi Xiao[2], Qiwen Pan[1], Dongdan Chen[1], Jianrong Qiu[3], Guoping Dong[1]*

[1]State Key Laboratory of Luminescent Materials and Devices and Guangdong Provincial Key Laboratory of Fiber Laser Materials and Applied Techniques, School of Materials Science and Engineering, South China University of Technology, Guangzhou 510640, China

[2]Key Laboratory of Renewable Energy, Guangdong Key Laboratory of New and Renewable Energy Research and Development, Guangzhou Institute of Energy Conversion, Chinese Academy of Sciences, Guangzhou 510640, China

[3]College of Optical Science and Engineering, State Key Laboratory of Modern Optical Instrumentation, Zhejiang University, Hangzhou 310027, China

*Correspondence to [dgp@scut.edu.cn (G. Dong)]

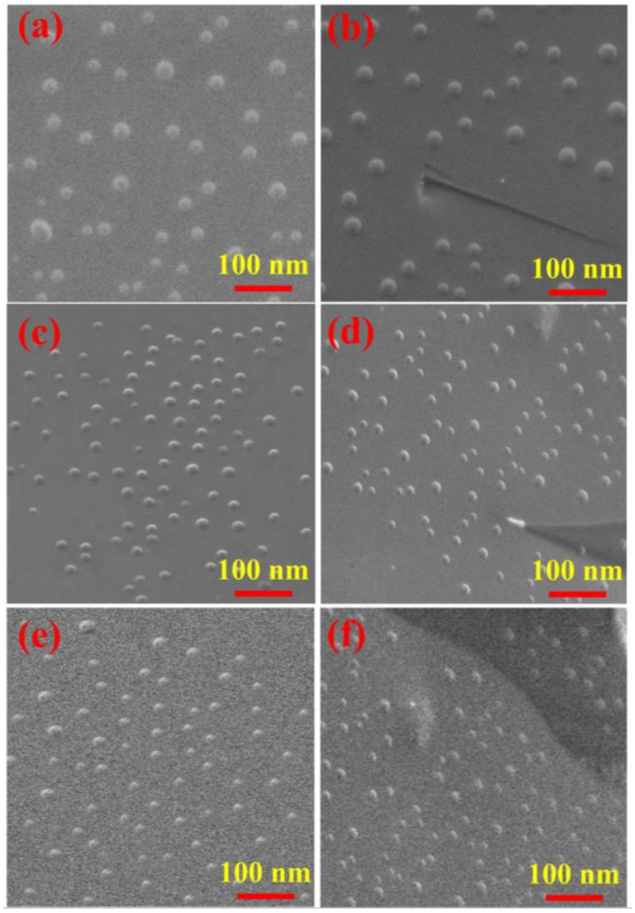

Figure S1. SEM images of the surface and section of (a)-(b) $Te_{59}Ge_{0}$ GC heat-treated at 430°C for 8 h, (c)-(d) $Te_{29}Ge_{30}$ GC heat-treated at 520°C for 8 h, and (e)-(f) $Te_{0}Ge_{59}$ GC heat-treated at 610°C for 8 h. It can be seen that the nanocrystals precipitated in both the surface and section, indicating the crystallization is a bulk process.

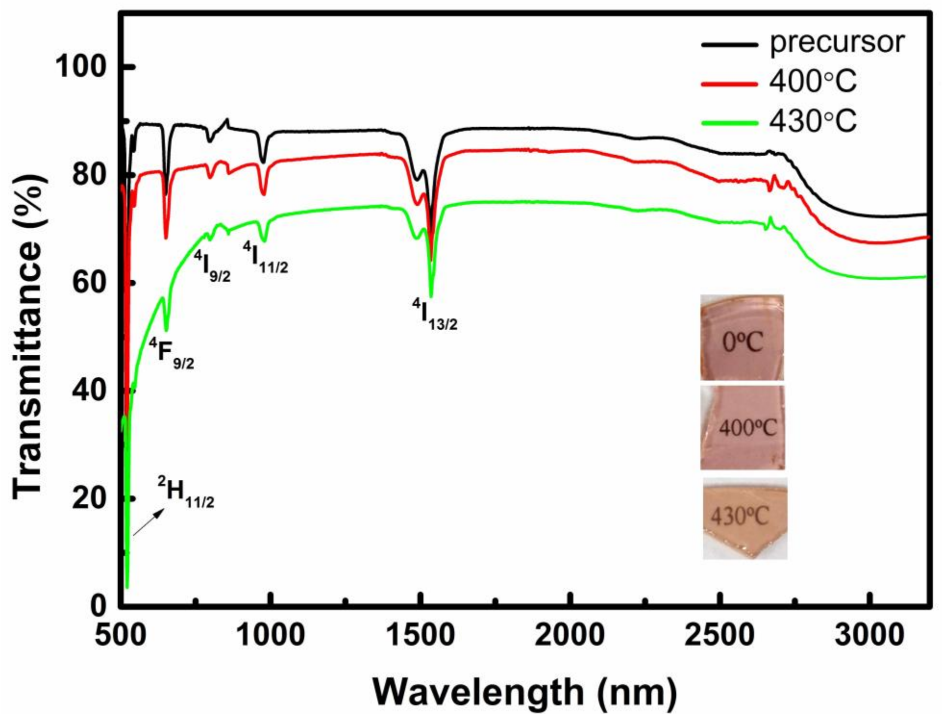

Figure S2. Transmittance spectra of $Te_{59}Ge_0$ glass and GCs. Inset shows the images of precursor glass and GCs with 1.5 mm thickness. It is observed that the transmittance of GCs is ~70% around 2.7 μm when the heat-treated temperature is below 430ºC. The inset of Fig. S2 shows that $Te_{59}Ge_0$ glass still has a good transparency after heat treatment.
